# Quantitative Analysis of Temperature Dependence of Raman shift of monolayer WS_**2**_

**DOI:** 10.1038/srep32236

**Published:** 2016-08-31

**Authors:** Xiaoting Huang, Yang Gao, Tianqi Yang, Wencai Ren, Hui-Ming Cheng, Tianshu Lai

**Affiliations:** 1 State Key Laboratory of Optoelectronic Materials and Technologies, School of Physics, Sun Yat-sen University, Guangzhou 510275, China; 2 Shenyang National Laboratory for Materials Science, Institute of Metal Research, Chinese Academy of Sciences, 72 Wenhua Road, Shenyang 110016, China

## Abstract

We report the temperature-dependent evolution of Raman spectra of monolayer WS_**2**_ directly CVD-grown on a gold foil and then transferred onto quartz substrates over a wide temperature range from 84 to 543 K. The nonlinear temperature dependence of Raman shifts for both 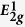 and A_**1g**_ modes has been observed. The first-order temperature coefficients of Raman shifts are obtained to be −0.0093 (cm^**−1**^/K) and −0.0122 (cm^**−1**^/K) for 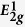 and A_**1g**_ peaks, respectively. A physical model, including thermal expansion and three- and four-phonon anharmonic effects, is used quantitatively to analyze the observed nonlinear temperature dependence. Thermal expansion coefficient (TEC) of monolayer WS_2_ is extracted from the experimental data for the first time. It is found that thermal expansion coefficient of out-plane mode is larger than one of in-plane mode, and TECs of 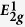 and A_**1g**_ modes are temperature-dependent weakly and strongly, respectively. It is also found that the nonlinear temperature dependence of Raman shift of 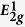 mode mainly originates from the anharmonic effect of three-phonon process, whereas one of A_**1g**_ mode is mainly contributed by thermal expansion effect in high temperature region, revealing that thermal expansion effect cannot be ignored.

Since the discovery of graphene, atomically thin two-dimensional (2D) layered materials have drawn intense attention due to their unique physical, chemical, and mechanical properties^1^,^2^. 2D transition metal dichalcogenides (TMDCs) such as MX_2_ (M = Mo, W and X = Se, S) have been successfully synthesized and prove to be one of the most stable atomically thin 2D materials^1^,^3^. Few layer TMDCs are stacked by several monolayers interacted by weak Van der Waals forces. Monolayer TMDCs consist of a triangular or hexagonal plane of transition metal M atom sandwiched by two triangular layers of dichalcogenides X atom^4^. Unlike graphene, monolayer TMDCs don’t have inversion symmetry of crystal space group so that TMDCs undergo a transition of band structures from an indirect band gap in bulk to a direct band gap in a monolayer material^5^, making them useful in nanoelectronic device applications^6^,^7^,^8^. For example, monolayer WS_2_ has a direct band gap of 2.1 eV, while the bulk WS_2_ has an indirect gap at 1.3 eV^9^. Among all TMDCs, monolayer MoS_2_ was first synthesized and has been studied extensively. Its field-effect transistors (FET) have been demonstrated and exhibited large current on/off ratio^10^. In contrast to MoS_2_, WS_2_ was synthesized later and studied much less. It was reported recently that monolayer WS_2_ had intense photoluminescence demonstrating the characteristics of its direct band gap^11^ and giant spin-valley coupling^12^, which implied potential applications in light emission, optical sensors and spin valleytronics applications^1^,^2^.

The integrated photoelectronic devices based on monolayer WS_2_ will have extremely high degree of integration, and hence heat transfer and phonon behaviors are very important for heat management of nanointegrated devices. Raman spectroscopy is a powerful tool and has been used widely to non-destructively characterize the structure, symmetries, and optical phonon behaviors of nanomaterial. The position and width of Raman scattering peak can reflect vibrational frequency and dynamics of optical phonons, respectively, while the latter is directly related to the heat diffusion rate. In nanoelectronic device application, it is very important to understand the effect of phonons because the self-heating of the device can significantly affect the performance. The temperature dependence of Raman shift of monolayer and few layer MoS_2_ has been extensively studied over a heating process^13^,^14^, a cooling process^15^, and a wide temperature range from 77 K to 623 K^16^. Thermal conductivity of MoS_2_ was given out based the temperature dependence of Raman shifts^16^,^17^. The effect of different-type substrates on temperature dependence of Raman shifts was also reported from room temperature to 500 °C^18^. In contrast, temperature dependence on Raman shifts of WS_2_ was reported sparsely^3^,^19^,^20^,^21^,^22^, so that thermal conductivity of monolayer WS_2_ was reported sparsely too^20^, and thermal expansion coefficient has not been reported yet. Thripuranthaka *et al*. reported the first experimental investigation on temperature-dependent Raman shifts of mechanically exfoliated monolayer WS_2_ transferred onto a Si substrate over a wide temperature range from 77 K to 623 K^19^, and observed an obvious nonlinear temperature dependence of Raman shifts of 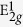 and A_1g_ modes. However, authors ignored the nonlinear dependence, and gave out a small first-order temperature coefficient of −0.006 cm^−1^/K for both 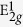 and A_1g_ modes by simply linear fitting to temperature-dependent Raman shifts^19^. Peimyoo *et al*. studied temperature dependent Raman shifts of mono- and bi-layer WS_2_ grown on Si substrates by chemical vapor deposition (CVD) over a low temperature range from 80 to 380 K^20^, but observed a good linear temperature dependence of Raman shifts of 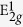 and A_1g_ modes, and gave out a large first-order temperature coefficients of −0.0125 and −0.0149 cm^−1^/K, respectively for 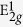 and A_1g_ modes^20^. Su *et al*. studied the temperature dependence of Raman shifts of CVD-grown monolayer WS_2_ films onto SiO_2_/Si and sapphire substrates as well as transferred on SiO_2_ substrates over a high temperature range from 25 to 500 °C^21^, and found good linear temperature dependences of Raman shift of 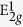 mode of all samples except for the WS_2_ grown on SiO_2_/Si substrates, but complex nonlinear temperature dependences of Raman shift of A_1g_ mode of all sample, showing strong dependence of Raman shifts on substrate types and bonding between WS_2_ and substrates. Gaur *et al*. studied the temperature dependence of Raman shifts of CVD-grown monolayer WS_2_ on Al_2_O_3_ substrates over a wide range from 83 to 573 K^22^, and observed a weakly nonlinear temperature dependence of Raman shift of A_1g_ mode. Those reports mentioned above presented diverse temperature dependences on Raman shift of 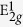 and A_1g_ modes, or inconsistent temperature dependences to each other. On one hand, the diverse temperature dependences may show strong dependence of Raman shift on sample-prepared methods, substrate types and bonding between WS_2_ sample and substrates. Meanwhile, it was also implied that the reliability of temperature dependences reported may need to be confirmed further, and hence more experimental studies are necessary very much to extract the accurate temperature coefficient of Raman shift of Raman active modes because the temperature coefficient directly reflects the strength change of Raman vibration bond with varying temperature. It is also an important parameter to differentiate layer number of layered films. Moreover, quantitative analysis of temperature dependence of Raman shifts using physical models is also absent so that physical origin of nonlinear temperature dependence is not clear. Even the experimental value of the thermal expansion coefficient of monolayer WS_2_ has not been available so far so that some theoretically calculated values of the in-plane modes for monolayer WS_2_^23^,^24^,^25^ could not be verified experimentally. Moreover, experimentally the only reported values of thermal expansion coefficients of bulk 2H-WS_2_ are also puzzled because the value of in-plane mode was as twice more as one of out-plane mode^26^.

In this article, we investigate temperature dependence on Raman shift of monolayer WS_2_^27^, directly CVD-grown on a gold foil and then transferred onto quartz substrates over a wide temperature range from 84 to 543 K. To our knowledge, temperature dependence of Raman shift of monolayer WS_2_ with the combination of such a sample and substrate is studied for the first time. The nonlinear temperature dependence of Raman shifts for both 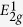 and A_1g_ modes has been observed. A physical model, including thermal expansion and three- and four-phonon anharmonic effects, is used quantitatively to analyze the observed nonlinear temperature dependence. Thermal expansion coefficient of monolayer WS_2_ is extracted from the experimental data for the first time. It is found that thermal expansion coefficient of out-plane mode is larger than one of in-plane mode, being more reasonable physically. It is also found that the nonlinear temperature dependence of Raman shift of 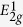 mode mainly originates from the anharmonic effect of three-phonon process, whereas one of A_1g_ mode is mainly contributed by thermal expansion effect in high temperature region, but still by three-phonon anharmonic effect in low temperature range. However, thermal expansion effect was ignored in the most of current reports^20^,^21^,^22^, obviously being not justified.

## Results

**Figure 1:**
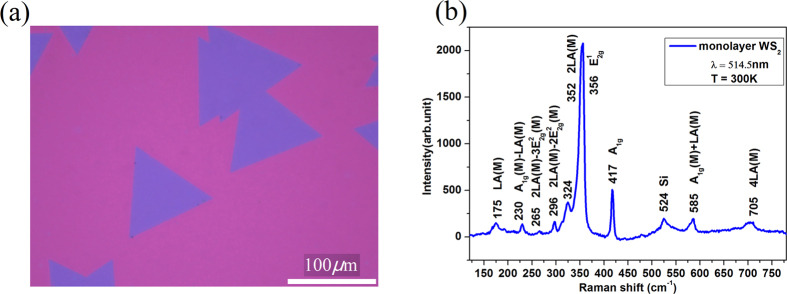
(**a**) Optical image of monolayer WS_2_ grown by CVD on gold foils transferred onto a SiO_2_/Si substrate for good optical contrast. (**b**) A typical Raman spectrum of the monolayer WS_2_ on a quartz substrate.

Monolayer WS_2_ sample studied here was grown on gold foil substrates by CVD, and then transferred onto a quartz substrate for Raman measurement and a SiO_2_/Si substrate for good optical contrast (See Fig. 1(a)). The details of the synthesis and crystal-quality characterization of monolayer WS_2_ can be found in ref. 27. Large area high-quality monolayer WS_2_ was grown by this CVD method on gold foils. As shown in Fig. 1(a), single crystal monolayer WS_2_ on SiO_2_/Si presents homogenous triangular blue domain and has a size over 100 μm. Here Raman spectroscopy is used further to characterize the quality and layer number of WS_2_ on quartz. The monolayer WS_2_ sample on a quartz substrate is mounted in a cryostat cooled by liquid nitrogen for measurement of temperature-dependent Raman shift.

A representative Raman spectrum over a wavenumber range of 100–800 cm^−1^ at room temperature is taken using 514.5 nm laser and shown in Fig. 1(b). The Raman spectrum consists of many first-order and second-order peaks^15^,^20^. The first-order peaks mainly include ones of 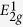 at 356 cm^−1^, A_1g_ at 417 cm^−1^, LA(M) at 175 cm^−1^ and the peak at 524 cm^−1^ tentatively assigned to Si-related microstructure in SiO_2_ substrates. The second-order peaks include ones of A_1g_ (M) − LA(M) at 230 cm^−1^, 2LA(M) − 3 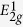 (M) at 265 cm^−1^, 2LA(M) −2E^2^ − 2 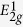 (M) at 296 cm^−1^, 2LA(M) at 352 cm^−1^, A_1g_(M) + LA(M) at 585 cm^−1^, and 4LA(M) at 705 cm^−1^. The positions of all peaks are extracted by multiple-peak Lorentzian fitting. The 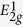 mode represents the in-plane vibrations of tungsten and sulfur atoms, and A_1g_ is associated with the out-of-plane vibrations of the sulfur atoms. The vibrational frequency of 2LA(M) and 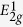 modes is close to each other and their Raman peaks overlap with each other. The asymmetric shape of 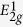 peak is the characteristic of double-peak overlapping. We fit the overlapping peak around 354cm^−1^ with double Lorentzian sum function to separate Raman peak of 2LA(M) from 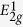 modes, showing up that Raman peak of 2LA(M) mode is much stronger than one of 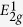 mode in intensity due to double resonance characteristic of 2LA(M) mode. Raman frequency difference between 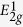 and A_1g_ modes is found less than 61 cm^−1^, indicating that the sample is monolayer^28^,^29^.

**Figure 2:**
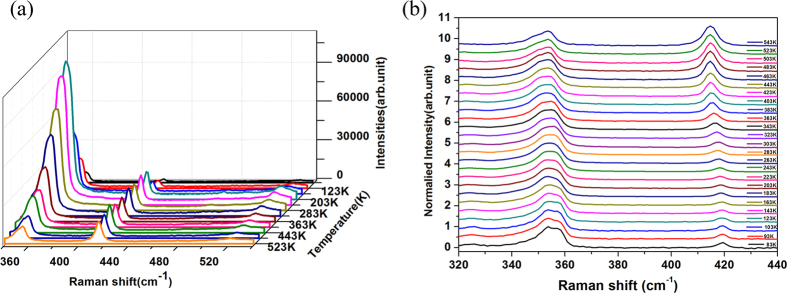
(**a**) Raman spectra of monolayer WS_2_ Raman modes in the range of 340–540 cm^−1^ from 83 K to 543 K. (**b**) Raman spectra normalized by the maximum intensity of Raman peak containing 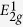 mode over 83 K to 543 K.

The temperature-dependent Raman spectra of monolayer WS_2_ are taken over a wide temperature range from 83 K to 543 K, and are plotted in Fig. 2(a) in the form of the waterfall graph to show the variation of peak intensity clearly with the temperature, where only Raman peaks of phonon modes that we are interesting in, are displayed in the range of 340–540cm^−1^ including 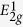, A_1g_ and Si-related peaks. One can find two obvious features. One is that the combined peak of 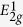 and 2LA modes is much stronger than A_1g_ peak in amplitude. The other is significantly non-monotonous variation of the amplitude of the combined peaks at ~354 cm^−1^ with temperature. Similar features were also reported^21^,^22^. the first feature originates from the significant enhancement of 2LA(M) mode under the excitation of 514.5 nm laser whose energy is in the vicinity of B exciton of WS_2_^30^,^31^, as shown similarly in refs 21 and 22. The second feature originates from temperature dependence of B exciton energy of monolayer WS_2_^32^,^33^. The energy of B exciton is almost resonant to photon energy of 514.5 nm laser as the temperature of WS_2_ is set at ~223 K, so that Raman scattering intensity approaches maximum. As temperature deviates from 223 K, the energy of B excitons becomes non-resonant to the photon energy of incident laser so that Raman scattering intensity weakens. The more far the deviation of temperature is from 223 K, the larger the detune is between the energies of B exciton and laser photon, and hence the weaker the Raman scattering intensity is. However, there is a difference of 70 K between this work and ref. 32. for the temperature that strongest Raman scattering peaks appeared. It may just reflect the effect of different substrates and bonding between film and substrates^31^.

In order clearly to display the shift of Raman peak position with temperature, the Raman spectra normalized by the intensity of overlapped peak of 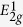 and 2LA(M) modes are plotted in Fig. 2(b). One can easily discern the red-shift of all Raman peaks with increasing temperature, similar to what was observed for MoS_2_ monolayers^13^,^14^,^15^,^16^,^17^,^18^. One can also find that the contribution of 2LA(M) mode in the wide overlapped peak of 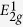 and 2LA(M) modes becomes weak with the increase of temperature, while one of 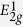 mode is enhanced.

## Discussion

**Figure 3:**
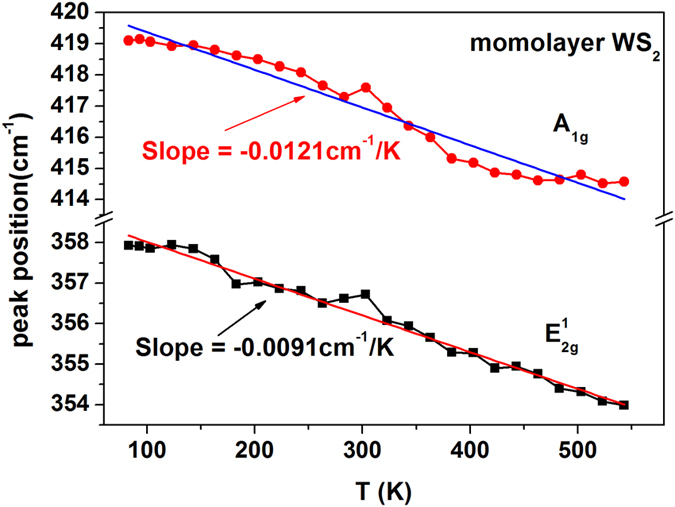
Raman shifts of 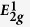 and A_1g_ modes as a function of temperature. The linear fit to experimental data and slope values are shown.

Multiple-peak Lorentzian sum function is used to best fit each Raman spectrum. The peak positions of multiple modes, including 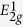, 2LA(M) and A_1g_ modes, are extracted simultaneously. The peak position of 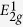 and A_1g_ modes that we are focusing on are plotted in Fig. 3 as a function of the lattice temperature of monolayer WS_2_. One can see that the temperature dependence of Raman shift of 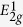 mode is weakly nonlinear, while one of A_1g_ mode is strongly nonlinear.

The temperature dependence of Raman shifts of 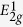 and A_1g_ modes is preliminarily analyzed by a linear approximation. The following linear equation is used best to fit the temperature dependence in Fig. 3, as done in refs 19, 20, 21.


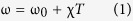
where ω_0_ is the extrapolated peak position at zero Kelvin, and χ is the first-order temperature coefficient. The best linear fitting is plotted in Fig. 3 by solid lines. It gives out the first-order temperature coefficients of monolayer WS_2_ as −0.0093 (cm^−1^/K) and −0.0122 (cm^−1^/K), respectively for 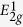 and A_1g_ modes, which are well in the range of the minimum (−0.0066 cm^−1^/K^19^) and maximum (−0.0155 cm^−1^/K^21^) reported for 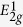 mode, and one of the minimum (−0.006 cm^−1^/K^19^) and maximum (−0.0149 cm^−1^/K^20^) reported for A_1g_ mode, respectively. Therefore, our results provide new reference data for first-order temperature coefficients of monolayer WS_2_.

One can see from Fig. 3 the temperature dependence actually is nonlinear, similar to previous reports^19^,^21^,^22^. However, main origin of the nonlinear dependence has been unknown yet because previous reports either ignored the nonlinear dependence^19^ or simply analyzed it with cubic polynomial^21^. Gaur *et al*. made an only physical analysis with anharmonic model, but ignored thermal expansion effect, so that the dominant origin of the nonlinear dependence was still unclear, being thermal expansion or anharmonic effects, or both of them cooperatively? Here we make the first quantitative analysis using a full model including both thermal expansion and anharmonic effects. As a result, thermal expansion coefficient of monolayer WS_2_ is obtained for the first time.

Before the quantitative analysis is started, it is very necessary to discuss the temperature dependence of A_1g_ mode shown in Fig. 3 because it looks quite strange. The Raman shift reduces and the reduction rate increases progressively with increasing temperature in the range of below ~380 K. However, such a change trend stops at ~380 K. Then a nearly linear slow decrease starts from 380 up to 460 K. Finally, the dependence becomes almost unchanged in the range of 460 to 550 K. Such temperature dependence of A_1g_ mode is very similar to ones observed by Su *et al*. on mechanically exfoliated and CVD-grown monolayer MoS_2_^18^ and CVD-grown WS_2_^21^ transferred onto SiO_2_/Si substrates, where anomalous temperature-dependent change occurred at ~100 °C (corresponding 373 K, agreeing very well with 380 K here). The anomalous temperature dependence of A_1g_ mode starting at ~380 K may be explained by possible forming of wrinkles or ripples in monolayer WS_2_ because thermal expansion coefficient of WS_2_ is about one order of magnitude higher than that of SiO_2_^21^, whereas the wrinkles or ripples can lead to significant strain in monolayer WS_2_ and weakening of bonding between the sample and the substrate. It was reported that the out-of-plane mode (A_1g_) was much more sensitive to and stronger affected by the bonding between the film and the substrate than the in-plane mode (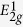)^21^, so that wrinkles or ripples results in anomalous temperature dependence of Raman shift of A_1g_ mode in high temperature range, but not one of 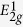 mode. To avoid the effect of significant strain on the temperature dependence, we analyze quantitatively the temperature dependence of A_1g_ mode only in the range of below 380 K, but one of 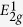 mode in whole range of 83–543 K.

To understand the physical origin of these nonlinear temperature dependencies, a physical model, including thermal expansion and three- and four-phonon anharmonic effects^18^,^34^, is used to quantitatively analyze the nonlinear temperature dependence of Raman shifts of 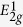 and A_1g_ modes. The model can be expressed as^18^,


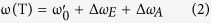
where 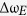 and 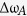 are Raman shift change induced by lattice thermal expansion and pure temperature effects, respectively. Volume expansion-induced contribution to the change of Raman shift can be described by Grüneisen constant model^18^,


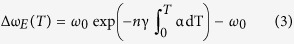
where n is the degeneracy, 1 for A_1g_ mode and 2 for 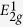 mode, γ_G_ is the Grüneisen parameter, and α is the thermal expansion of the material. The integration denotes the decrement of the vibrational frequency resulting from the expansion of volume. As the Grüneisen parameter (γ_G_) and thermal expansion coefficient (α) of monolayer WS_2_ material is unknown experimentally in our wide temperature range, we write their product as a polynomial expression,


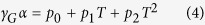
where *p*_0_, *p*_1_ and *p*_2_ are constants, and will be obtained as fit parameters by best fitting to the nonlinear temperature dependence.

The contribution from pure temperature effect mainly considers the anharmonic effects of three- and four-phonon processes. According to the viewpoint of Klemens^34^, the light scattering process can be viewed as involving the absorption of a photon, the emission of a photon, and the creation of an optical phonon which then decays via anharmonicity into two phonons, three phonons, etc. The production of two and three phonons is called three-phonon processes and four phonon processes, respectively. The pure temperature effect including three- and four-phonon processes can be described by a semi-quantitative simple model developed by Klemens^34^,


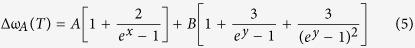
where 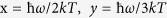, and coefficients A and B are constants as fit parameters, representing the contributions of three- and four-phonon processes to the frequency shift, respectively.

**Figure 4:**
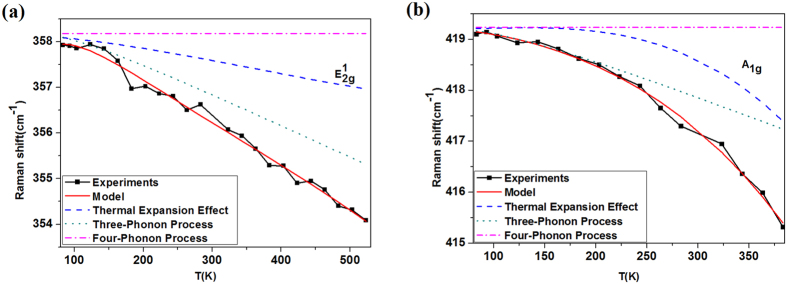
(**a**) The modelling of temperature dependence of Raman shift (solid line) and the individual contribution from thermal expansion (marked by dash), three-phonon (marked by dot) and four-phonon (marked by dash dot) processes as compared to the experimental results (scattered filled squares) of monolayer WS_2_ in (**a**)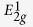 and (**b**) A_1g_ modes.

Equations (2, 3, 4, 5) are used best to fit the nonlinear temperature dependence of Raman shifts shown in Fig. 3. The fits are plotted in Fig. 4 by red solid lines and agree very well with experimental point data. Meanwhile, individual contribution of thermal expansion, three- and four-phonon effects is also plotted in Fig. 4. All fit parameters are extracted as A = −0.902 ± 0.047, B ≈ 0., *p*_0_ = (1.619 ± 2.467) × 10^−6^, *p*_1_ = (3.523 ± 2.893) × 10^−8^ and *p*_2_ = −(4.751 ± 6.051) × 10^−11^ for 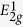 mode and A = −1.200 ± 0.025, B ≈ 0., *p*_0_ = (3.471 ± 2.462) × 10^−6^, *p*_1_ = −(9.952 ± 3.922) × 10^−8^ and *p*_2_ = (5.527 ± 1.103) × 10^−10^ for *A*_1g_ mode. It is worth noting that actually only four free fit parameters exist in our fit model because parameter B is found very small so that it may be set to zero and ω_0_ can be determined priorly by nonlinear fitting. The uncertainties of parameters A and (*p*_0_, *p*_1_, *p*_2_) are given only if one of the two group parameter is fixed to the mean. The allowed uncertainty of parameters, *p*_0_, *p*_1_ and *p*_2_, is larger because error mainly occurs in high temperature range, and is not distributed homogenously in the whole experimental temperature range.

One can see that the dominant contribution to nonlinear temperature-dependent Raman shift of planar mode 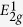 is from the three-phonon anharmonic process, while thermal expansion contributes weakly and the contribution of four-phonon anharmonic effect is completely negligible. In contrast, for A_1g_ mode, the contribution of thermal expansion and three-phonon anharmonic effects competes with each other though four-phonon process is negligible. In low temperature range, the latter is dominant, while the former is dominant in high temperature region. Our analysis reveals the quantitative contribution of thermal expansion, three- and four-phonon effects for the first time. The contribution of thermal expansion cannot be ignored for either *A*_1g_ or 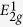 mode.

**Figure 5:**
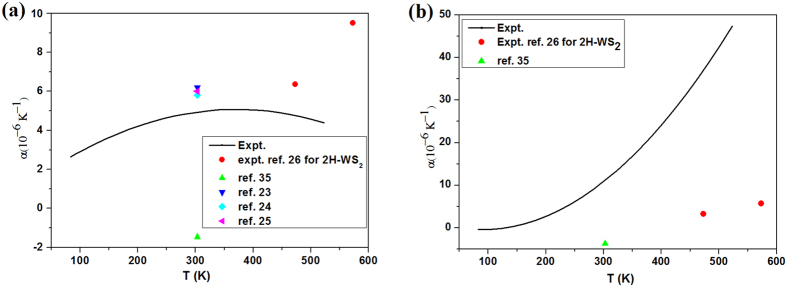
(**a**) Temperature-dependent thermal expansion coefficients of (**a**)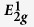 and (**b**) A_1g_ modes. The deduced experimental result is marked by black solid line. The red circle is the experimental result in ref. 23. The others are the theoretical calculated value obtained from the reference literature in 300 K.

The parameters, *p*_0_, *p*_1_ and *p*_2,_ have been extracted in last section. Instituting them into Eq. (4), the temperature dependence of the product of thermal expansion coefficient and Grüneisen parameter, γ_G_α, can be achieved. If the theoretical calculated value of Grüneisen parameter^35^, γ_G_ (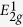) = 0.9176 and γ_G_ (A_1g_) = 2.1707, are adopted, the temperature dependence of thermal expansion coefficient (α) is obtained and plotted in Fig. 5. Meanwhile, several theoretical calculation^23^,^24^,^25^,^35^ and only experimental^26^ values reported are also plotted in Fig. 5 by scattered points. One can see that the thermal expansion coefficient of 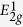 mode agrees well with the reported values in refs 23, 24, 25, 26 except for a negative value reported in ref. 35 (see Fig. 5(a)), whereas one of A_1g_ mode also agrees with three reported values and have the same temperature-dependent trend although it is significantly larger than reported ones (see Fig. 5(b)). More important is in our results that the values of 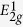 mode is smaller than ones of A_1g_ mode above room temperature, but in unique experimental report in ref. 26 the values of 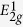 mode is larger than ones of A_1g_ mode. We believe our results may be more justified physically than ones in ref. 26 because in layered materials the out-of-plane direction is confined very weakly. Furthermore, our results show fully positive values above 130 K, which is reverse fully with ones in ref. 35.

In summary, we have reported temperature dependent Raman study of the first-order 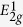 and A_1g_ mode in monolayer WS_2_ sample directly CVD-grown on a gold foil and then transferred onto quartz substrates over a wide temperature range from 84 to 543 K. The nonlinear temperature dependence of Raman shifts for both 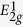 and A_1g_ modes has been observed. The first-order temperature coefficients of Raman frequency shifts are given as −0.0093 (cm^−1^/K) and −0.0122 (cm^−1^/K) for 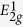 and A_1g_ peaks, respectively. A physical model, including thermal expansion and three- and four-phonon anharmonic effects, is used quantitatively to analyze the observed nonlinear temperature dependence. Thermal expansion coefficient of monolayer WS_2_ is extracted from the experimental data for the first time. It is found that thermal expansion coefficient one of out-plane mode is larger than one of in-plane mode, being more reasonable physically. It is also found that the nonlinear temperature dependence of Raman shift of 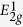 mode mainly originates from the anharmonic effect of three-phonon process, whereas one of A_1g_ mode is also mainly contributed by three-phonon process in low temperature range but by thermal expansion effect in high temperature region. Our results are useful for further experimental and theoretical studies on the thermal properties of two dimensional materials and the development of nano devices of WS_2_. They are also helpful to get deep insight into heat transfer and photon dynamics of monolayer WS_2_.

## Methods

Monolayer WS_2_ sample studied here was grown on gold foil substrates by CVD, and then transferred onto a quartz substrate. The details of the synthesis and crystal-quality characterization of monolayer WS_2_ can be found in ref. 27. Large area high-quality monolayer WS_2_ could be grown by this CVD method on gold foils. Single crystal monolayer WS_2_ is triangular, and has a size over 100 μm.

The micro-Raman system used in this study is Renishaw inVia with a Linkam TS1500 heating system. Renishaw inVia Micro-Raman system has a spectral resolution smaller than 1 cm^−1^ and a 50 × long working-distance lens which can focus laser beam to a spot less than 1 μm in diameter. A 514.5 nm laser is used. The power incident on the sample is 1.97 mW, low enough to avoid heating of the sample. The Linkam TS1500 heating system has a temperature control accuracy of 1 °C, heating the sample with a step of 10 °C at a rate of 10 °C/min. To stabilize the sample temperature, ten minutes’ delay is applied at each temperature step till a Raman spectrum is taken, ensuring sufficient time to reach thermal equilibrium.

## Additional Information

**How to cite this article**: Huang, X. *et al*. Quantitative Analysis of Temperature Dependence of Raman shift of monolayer WS_2_. *Sci. Rep.* **6**, 32236; doi: 10.1038/srep32236 (2016).
